# The Association between Diffuse Myocardial Fibrosis on Cardiac Magnetic Resonance T1 Mapping and Myocardial Dysfunction in Diabetic Rabbits

**DOI:** 10.1038/srep44937

**Published:** 2017-03-24

**Authors:** Mu Zeng, Yingyan Qiao, Zhaoying Wen, Jun Liu, Enhua Xiao, Changlian Tan, Yibin Xie, Jing An, Zishu Zhang, Zhanming Fan, Debiao Li

**Affiliations:** 1 Department of Radiology, The Second Xiangya Hospital of Central South University, Changsha, China; 2 Department of Ultrasound, The Second Hospital of Shanxi Medical University, Taiyuan, China; 3 Department of Radiology, Beijing An Zhen Hospital, Capital Medical University, Beijing Institute of Heart, Lung and Blood Vessel Disease, Beijing, China; 4 Biomedical Imaging Research Institute, Cedars-Sinai Medical Center, Los Angeles, California, USA; 5 MR Collaborations NE Asia, Siemens Healthcare, Beijing, China

## Abstract

The objective of this study was to assess the relationship between imaging surrogates for diffuse fibrosis and myocardial dysfunction. Thirty-six New Zealand white rabbits were classified into two groups: a control group (n = 18) and an alloxan-induced diabetes mellitus (DM) group (n = 18). For all rabbits, conventional ultrasonography, two-dimensional speckle tracking, and cardiac magnetic resonance (CMR) T1 mapping were performed; all of the rabbits were then sacrificed for Masson’s staining. The extracellular volume (ECV) was calculated from pre- and post-contrast T1 values and compared with myocardial function measured by echocardiography using Pearson’s correlation. In the DM group, ECV increased as the duration of diabetes increased, consistent with the changes in myocardial fibrosis verified by pathology. Moreover, ECV was strongly correlated with the early diastolic strain rate (r = −0.782, p < 0.001) and moderately correlated with the radial systolic peak strain (r = 0.478, p = 0.045). Thus, ECV is an effective surrogate for myocardial diffuse fibrosis on CMR imaging, and higher ECV values are associated with an increased impairment of myocardial diastolic function.

Diabetes mellitus (DM) can result in structural cardiac changes and myocardial dysfunction, leading to heart failure^1^,^2^. A 9-year follow-up epidemiological study of patients who suffered from myocardial damage demonstrated that the morbidity and mortality rate of patients with diabetes is higher than that of patients without diabetes^3^. Although the potential pathogenesis of diabetic myocardial damage may be multifactorial^4^, accelerated cellular apoptosis and necrosis eventually occur, resulting in increased diffuse myocardial interstitial fibrosis^1^. Fibrosis may be contribute to myocardial dysfunction because of its potential associations with hyperglycemia^5^. However, myocardial dysfunction in the early stage may result from abnormal myocyte function and hypertrophy rather than from fibrosis^6^. Therefore, the relationship between fibrosis and myocardial dysfunction remains controversial.

Two-dimensional (2D) speckle tracking is an advanced, highly sensitive echocardiographic technique for the early detection of subtle diabetic myocardial dysfunction^7^. However, because of its technical limitations, the use of an integrated backscatter ultrasound technique for the detection of diffuse myocardial fibrosis has several limitations^8^. Cardiac magnetic resonance (CMR) T1 mapping has recently been developed and uses inversion recovery, saturation recovery, and Look-Locker methods. CMR T1 mapping has better spatial and temporal resolution and can noninvasively detect diffuse myocardial fibrosis^9^,^10^. Because different tissues have specific ranges of T1 values at a particular magnetic field strength, CMR T1 mapping can quantify the degree of fibrosis by accurately measuring the extracellular volume (ECV), which is calculated from pre- and post-contrast T1 values^11^,^12^.

Diabetic rabbits are known to exhibit myocardial fibrosis^13^,^14^. Therefore, in this study, we established a diabetic rabbit model and continuously observed the changes in cardiac function and the degree of diffuse interstitial fibrosis. Our hypothesis was that after the induction of diabetes, rabbits will develop diffuse myocardial fibrosis that can lead to myocardial dysfunction.

## Results

### Characteristics

The rabbits in the DM group gradually resumed their diet. In the DM group, 1 rabbit died within 8 hours after the alloxan injection, 3 rabbits died after model induction, and the blood glucose levels of 2 other rabbits gradually returned to normal. Thus, in the DM and control groups, a total of 36 rabbits were included in the analysis, and each subgroup contained 6 rabbits.

### Echocardiography

The morphology and function of the left ventricle (LV) were assessed via conventional echocardiography in both the DM and control groups (Table 1). No significant difference in the ejection fraction (EF) was observed between the two groups (p > 0.05). Additionally, 2D speckle tracking showed that, at 3 months, no difference in radial systolic peak strain (SR) (t = −0.535, p = 0.604) and early diastolic strain rate (SrR) (t = −0.260, p = 0.800) could be found between the two groups. At 6 months, there was still no difference in SR (t = 0.143, p = 0.889), but a significant difference in SrR (t = 2.401, p = 0.037) was evident between the two groups. Moreover, at 9 months, significant differences were identified in both SR (t = −5.052, p < 0.001) and SrR (t = 6.081, p < 0.001) between the DM and control groups.

### CMR and its correlation with echocardiography

**Figure 1:**
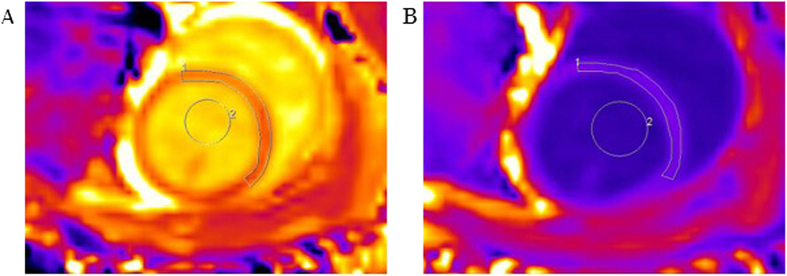
Representative T1 maps for a typical rabbit in the 6-months diabetes group. (**A**) Pre-contrast T1 map. ROI 1 shows the T1 pre-contrast myocardium time: 1,330 ± 86 ms, and ROI 2 shows the T1 pre-contrast blood time: 1,876 ± 27 ms. (**B**) Post-contrast T1 map from the same level. ROI 1 shows the T1 post-contrast myocardium time: 513 ± 16 ms, and ROI 2 shows the T1 post-contrast blood time: 408 ± 6 ms. Hematocrit: 42%. The ECV calculated from pre- and post-contrast T1 maps is 36.2%.

**Figure 2:**
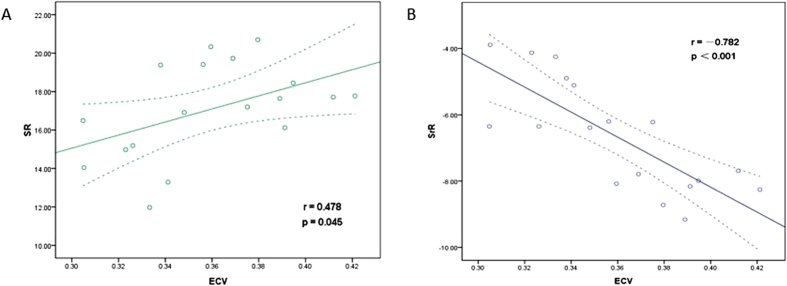
Correlations between myocardial fibrosis on MR images and cardiac systolic and diastolic function on ultrasonography images in the DM group. (**A**) Correlation between the ECV and SR; (**B**) Correlation between the ECV and SrR.

The CMR T1 mapping results are shown in Fig. 1. The ECV was calculated from the T1 values before and after contrast administration. The ECV differed significantly between the DM and control groups (t = 2.46, p = 0.034) at 3 months; this is earlier than the time point at which significant differences in SR and SrR could be detected. Significant differences in the ECV between the DM and control groups persisted at 6 months (t = 7.26, p < 0.001) and at 9 months (t = 9.89, p < 0.001). In the DM group, the ECV increased as the duration of diabetes increased and was strongly correlated with the SrR (r = −0.782, p < 0.001) and moderately correlated with the SR (r = 0.478, p = 0.045) (Fig. 2).

### Histology

**Figure 3:**
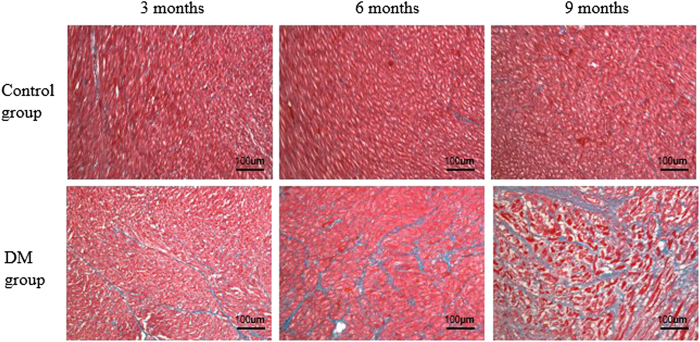
Histological assessment of myocardial alterations in accordance with the MR ROI areas in the interventricular septum myocardium. Masson’s staining (blue = fibrosis, red = myocardial cells) of the hearts of control and DM rabbits. Compared with the control group, the severity of diffuse interstitial fibrosis increased as the duration of diabetes increased in the DM group.

**Figure 4:**
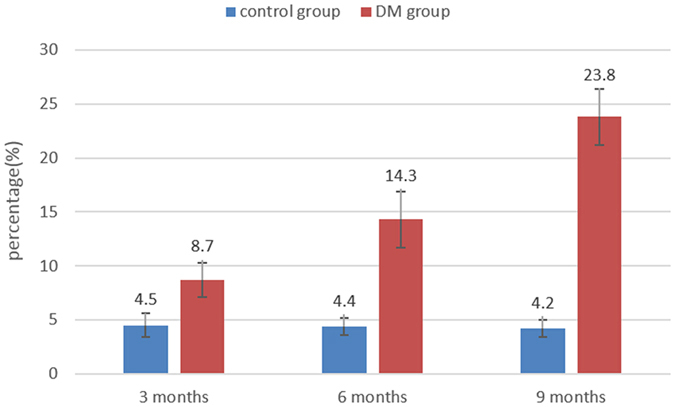
A bar plot presenting the CVF in the DM and age-matched control groups. CVF: collagen volume fraction; DM: diabetes mellitus.

Figure 3 shows the histological results of rabbit myocardium in the control and DM groups. In the DM group, as the duration of diabetes increased, more extensive myocardial fibrosis was observed. Figure 4 presents a bar plot of the collagen volume fraction (CVF) in the DM and age-matched control groups. The CVF values at 3, 6, and 9 months after model induction were 8.7 ± 1.6%, 14.3 ± 2.6%, and 23.8 ± 2.5%, respectively. In the control group, no significant change in myocardial fibrosis was observed from 3 to 9 months. The CVF values at 3, 6, and 9 months in the control group were 4.5 ± 1.1%, 4.4 ± 0.8%, and 4.2 ± 0.8%, respectively. The CVF differed significantly between the DM and control groups at all 3 time points (3 months: t = 5.28, p < 0.001; 6 months: t = 9.03, p < 0.001; and 9 months: t = 18.57, p < 0.001).

## Discussion

Although previous studies have shown that diabetes may induce myocardial dysfunction at an early stage, our study demonstrated the presence of diffuse myocardial fibrosis at different stages. We also established a correlation between fibrosis and myocardial dysfunction in diabetic rabbit models based on histological evaluation. Our main findings are as follows: (1) The changes in the ECV occurred earlier than those of other markers of cardiac dysfunction. (2) The ECV in diabetic rabbits is significantly higher than that in the control group, and ECV elevation was associated with the duration of rabbit diabetes. (3) The ECV was strongly correlated with diastolic dysfunction and moderately correlated with systolic dysfunction.

Diastolic dysfunction is the most frequent echocardiographic finding in patients with diabetes, even in those with a normal EF^15^,^16^. Animal experiments have revealed that rats and mice with streptozotocin (STZ)-induced diabetes exhibited impairments in diastolic function^17^,^18^. Although diastolic dysfunction has been observed at an early stage of diabetic heart disease in patients with normal LVEFs^19^, preclinical systolic alterations have recently been associated with strain^15^,^20^. An experimental rat study showed lower myocardial velocity and systolic strain rate and delayed time-to-peak deformation^21^. Another study demonstrated that 12 weeks after STZ induction, the systolic circumferential strain rates decreased mildly in diabetic rats^22^. Through the use of 2D speckle tracking, which is an advanced echocardiographic technique that is highly sensitive for the early detection of subtle myocardial dysfunction, we found that diabetes can lead to both diastolic and systolic dysfunction. According to our observations, the SrR first changed at 6 months and worsened at 9 months, whereas the SR first changed at 9 months. These findings indicate that diastolic dysfunction appeared earlier than systolic dysfunction and that diastolic function was increasingly impaired as the duration of diabetes increased. These findings were consistent with the results of previous studies.

CMR T1 mapping is a non-invasive technique for the quantification of diffuse myocardial interstitial fibrosis. A recent study by Arnold was designed to detect fibrosis in patients with diabetes with normal LVEF. Relative to healthy controls, the patients with diabetes had significantly lower post-contrast T1 values because of the increased burden of myocardial interstitial fibrosis^23^. A study conducted by Jellis showed that lower post-contrast T1 values were observed in patients with type 2 diabetes with abnormal insulin sensitivity^24^. The gadolinium contrast agent used could not pass through the cell membrane; thus, the post-contrast T1 value was primarily related to the contrast agent concentration outside the cell. However, multiple factors may contribute to this result, including the renal excretion rate, hematocrit, and acquisition time^25^; therefore, we chose to use the adjusted ECV, which is calculated from pre- and post-contrast T1 values, to represent fibrosis. The ECV ensures the balance of the contrast agent concentration between the myocardial extracellular space and the blood pool^26^. The ECV of the blood pool is represented as one minus the hematocrit value. Using this approach, the problems of post-contrast T1 mapping were fundamentally solved, and thus, the ECV can more accurately reflect changes in the myocardial extracellular space. In our study, we found that the change in the ECV occurred earlier than the change in the SrR, which suggests that the changes in cardiac morphology occur earlier than the changes in myocardial dysfunction. This finding demonstrates the advantage of CMR characteristic imaging, which can be used for the early detection of morphological changes. In the DM group, the ECV increased as the duration of diabetes increased, which indicates that a longer duration of diabetes was associated with a higher degree of myocardial fibrosis, consistent with the histological findings.

The most important finding of this study was that myocardial interstitial fibrosis was strongly correlated with diastolic dysfunction *in vivo*. Myocardial fibrosis and collagen deposition are the earliest morphological changes induced by DM and contribute to increased LV stiffness, which leads to LV dysfunction^1^. Previous studies showed that type 2 DM rats had a lower early diastolic peak velocity of the mitral valve. They also exhibited interstitial fibrosis based on histological examination, which suggests that LV fibrosis occurs early in type 2 diabetes^27^. Among patients with no clinical symptoms of diabetes, the results of ultrasonography backscattering for the evaluation of myocardial fibrosis suggest that patients with diabetes showed fibrosis at the ventricular wall and the spacer, which are closely observed in patients with late diastolic dysfunction^24^. The fibrotic areas are primarily distributed in the myocardial interstitium and peripheral blood vessels. Collagen can interact with the myocardial uptake of glucose to generate glycated collagen. Glycated collagen can further promote the glycosylation terminal product—advanced glycation-end products (AGEs)—which increase myocardial stiffness and accelerate changes in cardiac morphology and function^28^,^29^.

We successfully established the continuous diabetic rabbit model by dynamically monitoring the process of fibrosis *in vivo*. Pathology results confirmed that the ECV was an effective surrogate marker for the burden of myocardial diffuse fibrosis and that it was strongly correlated with diastolic dysfunction. One possible explanation is that AGEs led to myocardial stiffness by increasing the fibrosis collagen^30^. Systolic dysfunction is less strongly correlated with fibrosis than diastolic dysfunction, most likely because of the increased AGEs in the setting of hyperglycemia, which not only alters the extracellular matrix composition but also affects enzymatic activity and myocardial cell metabolism, thereby impairing myocardial cell exercise capacity^31^.

## Conclusions

The ECV is an effective CMR imaging surrogate for myocardial diffuse fibrosis. Higher ECV values are associated with a more severe impairment of myocardial diastolic function in diabetic rabbits.

## Methods

### Experimental model

All experiments involving rabbits were performed in accordance with the national guidelines for the use of experimental animals. The protocols were approved by the Ethics Committee of Laboratory Animals at the Capital Medical University of China. A total of 42 one-year-old male New Zealand white rabbits with a mean weight of 2.5 kg were used, and all animals were provided by the Animal Experiment Center of Clean Grade of Beijing Anzhen Hospital. The laboratory room temperature ranged from 18 °C to 25 °C, and the relative humidity ranged from 40% to 60%. One rabbit was reared in each cage. Ordinary fodder and water were provided. The rabbits were randomly divided into two groups: the DM group (n = 24) and the control group (n = 18). The DM group was subdivided into three groups: 3-month diabetes group (n = 8), 6-month diabetes group (n = 8), and 9-month diabetes group (n = 8). The control group was also subdivided into three groups to be consistent with the DM group: 3-month control group (n = 6), 6-month control group (n = 6), and 9-month control group (n = 6). After fasting for 12 hours, the rabbits in the DM group were first administered a single dose of alloxan (150 mg/kg) through an ear vein. Subsequently, they were administered 10% glucose intravenously over 12 hours to avoid insulin shock; a normal diet and oral fluid were provided. In the control group, normal saline administration with the same dose and rate of glucose administration were performed. The blood glucose levels of all rabbits were assessed weekly. For the rabbits in the DM group, we defined animals with fasting blood glucose values exceeding 16 mmol/L for more than 3 successive weeks as successful models.

### CMR imaging

A mixture of 1-mg/kg diazepam and 2.5-mg/kg xylazine hydrochloride was administered as anesthesia via an intramuscular administration to reduce the heart rate to nearly 100 beats/min. Before CMR imaging, blood samples were collected from each rabbit for hematocrit assessment. The chests of the rabbits were shaved for electrocardiogram (ECG) electrode placement. A commercial 8-Ch Rabbit Cardiac coil (Suzhou Medcoil Healthcare Co., Ltd.) was used, and all animals were scanned with a clinical 3-T scanner (Magnetom Verio, Siemens Healthcare, Erlangen, Germany). T1 quantification was performed with a Modified Look-Locker Inversion Recovery (MOLLI) prototype based on 8 images and 13 heartbeat 5-(5)-3 steady-state free precession (SSFP) sequences before and 15 min after the administration of 0.2-mmol/kg gadopentetate dimeglumine. The imaging parameters were as follows: field of view, 130 × 153 mm^2^; slice thickness, 4 mm; time of repetition (TR), 3.1 ms; echo time (TE), 1.3 ms; and matrix, 122 × 144 pixels. These parameters resulted in a spatial resolution of 1.1 × 1.1 mm^2^ and a flip angle of 35°. The acquisition time was approximately 8 s per slice. A region of interest (ROI) was defined in the LV myocardium to assess the pre- and post-myocardial T1 values. Another ROI was defined in the center of the LV blood pool to assess the pre- and post-contrast blood T1 values. The ECV was computed as follows: ECV = (1 − hematocrit) × (ΔR1 myocardium/ΔR1 blood), where R1 = 1/T1^11^. All data were manually analyzed using Siemens Syngo Argus commercial software.

### Echocardiography

After CMR scanning, transthoracic echocardiography (TTE) was performed with a Vivid7 Ultrasound cardiovascular system (GE Healthcare USA) using a 7–10 MHz transducer and an Echo PAC ultrasound workstation with STI imaging analysis software. Conventional measurements collected in the parasternal LV long-axis view using M-mode included the left atrial systolic diameter (LAd), LV end-diastolic diameter (LVIDd), LV end-systolic diameters (LVIDs), end-diastolic interventricular septum thickness (IVSd), and LV posterior wall thickness (LVPWd). LVEF was assessed using the biplane Simpson method from tracing images of the apical 4-chamber view and 2-chamber view. Routine grayscale 2D cine loops of 3 consecutive beats were obtained from the parasternal short-axis view of the LV at the level of the papillary muscle. The endocardial borders were traced in the end-systolic frame of the 2D images from the short-axis view to assess the LV radial myocardial strains with STI imaging analysis software. The SR and SrR were derived from the strain curve and strain rate curve in the following 6 segments: the septal, anteroseptal, anterior, lateral, posterior, and inferior walls. The strain curve and strain rate curve were obtained from the parasternal short-axis view of the LV at the level of the papillary muscle. The mean SR and SrR were defined as the mean value of each parameter in the 6 segments mentioned above.

### Histological analysis

After CMR and ultrasonography, the rabbits in both the DM and control groups were sacrificed. A solution of 10% formalin was used to fix the myocardium. After dehydration and embedding, pathological sections in the interventricular septum myocardium corresponding to the CMR scanning area were selected. The slice thickness was 5 μm, and the slices were subjected to Masson’s staining. After the artifacts and pericardial tissue were removed, we chose 12 light microscopic fields to calculate the CVF using the following formula: CVF = total collagen area/the total image area. We then calculated the average CVF of the collagen content of each slice.

### Statistical analysis

All data are expressed as the mean ± standard deviation. Differences in echocardiographic and CMR parameters between the DM and control groups were determined with a two-tailed unpaired Student’s t-test. The correlations between the ECV and SR or SrR were assessed using Pearson’s correlation analysis. SPSS 17.0 software was used for all statistical analyses. For all comparisons, p < 0.05 was considered statistically significant.

## Additional Information

**How to cite this article**: Zeng, M. *et al*. The Association between Diffuse Myocardial Fibrosis on Cardiac Magnetic Resonance T1 Mapping and Myocardial Dysfunction in Diabetic Rabbits. *Sci. Rep.* **7**, 44937; doi: 10.1038/srep44937 (2017).

**Publisher's note:** Springer Nature remains neutral with regard to jurisdictional claims in published maps and institutional affiliations.

## Figures and Tables

**Table 1:** Ultrasonography and CMR parameters.

	DM group				Control group			
	Baseline	3 months	6 months	9 months	Baseline	3 months	6 months	9 months
LA (mm)	9.56 ± 0.98	10.56 ± 0.58	9.97 ± 0.86	11.36 ± 0.82	9.95 ± 0.68	9.79 ± 0.81	10.61 ± 0.58	11.56 ± 0.67
IVS (mm)	2.32 ± 0.41	2.43 ± 0.32	2.42 ± 0.51	2.52 ± 0.62	2.63 ± 0.24	2.34 ± 0.32	2.75 ± 0.31	2.86 ± 0.63
LVPW (mm)	2.33 ± 0.51	2.56 ± 0.32	2.78 ± 0.67	2.89 ± 0.43	2.35 ± 0.19	2.67 ± 0.41	2.78 ± 0.53	2.89 ± 0.67
LVIDd (mm)	15.67 ± 0.59	16.67 ± 0.81	16.65 ± 0.78	17.67 ± 0.55	14.75 ± 0.87	15.68 ± 0.85	16.64 ± 0.54	16.98 ± 0.67
LVIDs (mm)	11.67 ± 1.21	12.87 ± 1.16	12.45 ± 1.13	12.67 ± 1.41	11.78 ± 1.32	11.97 ± 1.21	13.67 ± 1.29	12.67 ± 1.26
EF (%)	60.33 ± 4.51	61.33 ± 4.23	62.33 ± 3.57	62.67 ± 4.53	61.53 ± 4.23	63.33 ± 5.51	63.73 ± 4.35	62.37 ± 4.54
FS (%)	31.33 ± 3.16	32.33 ± 3.23	32.78 ± 3.45	34.33 ± 3.32	30.67 ± 3.15	31.54 ± 3.24	35.33 ± 4.51	34.33 ± 3.36
SR	18.63 ± 1.25	18.06 ± 1.50	18.83 ± 1.42	14.33 ± 1.60*	18.46 ± 1.37	18.54 ± 1.60	18.71 ± 1.33	18.35 ± 1.14*
SrR	−8.24 ± 0.64	−8.33 ± 0.53	−6.60 ± 1.17*	−5.01 ± 1.11*	−8.19 ± 0.72	−8.26 ± 0.42	−8.11 ± 1.01*	−8.16 ± 0.60*
ECV	29.96 ± 1.33	32.31 ± 1.54*	35.82 ± 1.41*	39.81 ± 1.63*	30.01 ± 1.21	30.05 ± 1.59*	29.83 ± 1.47*	30.33 ± 1.75*

*p < 0.05 vs. the control group at the same time point.
