# Author Correction: The Association Between Diffuse Myocardial Fibrosis on Cardiac Magnetic Resonance T1 Mapping and Myocardial Dysfunction in Diabetic Rabbits

**DOI:** 10.1038/s41598-022-14714-9

**Published:** 2022-06-15

**Authors:** Mu Zeng, Yingyan Qiao, Zhaoying Wen, Jun Liu, Enhua Xiao, Changlian Tan, Yibin Xie, Jing An, Zishu Zhang, Zhanming Fan, Debiao Li

**Affiliations:** 1 Department of Radiology, The Second Xiangya Hospital of Central South University, Changsha, China; 2 Department of Ultrasound, The Second Hospital of Shanxi Medical University, Taiyuan, China; 3 Department of Radiology, Beijing An Zhen Hospital, Capital Medical University, Beijing Institute of Heart, Lung and Blood Vessel Disease, Beijing, China; 4 Cedars-Sinai Medical Center, Biomedical Imaging Research Institute, Los Angeles, CA USA; 5 MR Collaborations NE Asia, Siemens Healthcare, Beijing, China

Correction to: *Scientific Reports* 10.1038/srep44937, published online 24 March 2017

This Article contains errors.

It does not specify that the cohort of rabbits used were the same as that in Qiao et al.^1^, and that, therefore, some of the data in Table 1 (including LA, IVS, LVPW, LVIDd, LVIs, EF, and FS) had already been published.

**Table 1:** Ultrasonography and CMR parameters.

	DM group				Control group			
	Baseline	3 months	6 months	9 months	Baseline	3 months	6 months	9 months
LA (mm)^1^	9.56 ± 0.98	10.56 ± 0.58	9.97 ± 0.86	11.36 ± 0.82	9.95 ± 0.68	9.79 ± 0.81	10.61 ± 0.58	11.56 ± 0.67
IVS (mm)^1^	2.32 ± 0.41	2.43 ± 0.32	2.42 ± 0.51	2.52 ± 0.62	2.63 ± 0.24	2.34 ± 0.32	2.75 ± 0.31	2.86 ± 0.63
LVPW (mm)^1^	2.33 ± 0.51	2.56 ± 0.32	2.78 ± 0.67	2.89 ± 0.43	2.35 ± 0.19	2.67 ± 0.41	2.78 ± 0.53	2.89 ± 0.67
LVIDd (mm)^1^	15.67 ± 0.59	16.67 ± 0.81	16.65 ± 0.78	17.67 ± 0.55	14.75 ± 0.87	15.68 ± 0.85	16.64 ± 0.54	16.98 ± 0.67
LVIDs (mm)^1^	11.67 ± 1.21	12.87 ± 1.16	12.45 ± 1.13	12.67 ± 1.41	11.78 ± 1.32	11.97 ± 1.21	13.67 ± 1.29	12.67 ± 1.26
EF (%)^1^	60.33 ± 4.51	61.33 ± 4.23	62.33 ± 3.57	62.67 ± 4.53	61.53 ± 4.23	63.33 ± 5.51	63.73 ± 4.35	62.37 ± 4.54
FS (%)^1^	31.33 ± 3.16	32.33 ± 3.23	32.78 ± 3.45	34.33 ± 3.32	30.67 ± 3.15	31.54 ± 3.24	35.33 ± 4.51	34.33 ± 3.36
SR	18.63 ± 1.25	18.06 ± 1.50	18.83 ± 1.42	14.33 ± 1.60*	18.46 ± 1.37	18.54 ± 1.60	18.71 ± 1.33	18.35 ± 1.14*
SrR	-8.24 ± 0.64	-8.33 ± 0.53	-6.60 ± 1.17*	-5.01 ± 1.11*	-8.19 ± 0.72	-8.26 ± 0.42	-8.11 ± 1.01*	-8.16 ± 0.60*
ECV	29.96 ± 1.33	32.31 ± 1.54*	35.82 ± 1.41*	39.81 ± 1.63*	30.01 ± 1.21	30.05 ± 1.59*	29.83 ± 1.47*	30.33 ± 1.75*

*p < 0.05 vs. the control group at the same time point.

As a result, in the Methods section, under the subheading ‘Experimental model’,

“A total of 42 one-year-old male New Zealand white rabbits with a mean weight of 2.5 kg were used, and all animals were provided by the Animal Experiment Center of Clean Grade of Beijing Anzhen Hospital.”

should read:

“The same cohort of 42 one-year-old male New Zealand white rabbits as that presented in Qiao et al.^1^ were used. The animals had a mean weight of 2.5 kg, and were provided by the Animal Experiment Center of Clean Grade of Beijing Anzhen Hospital.”

In addition, in the Results section, under the subheading ‘Echocardiography’,

“The morphology and function of the left ventricle (LV) were assessed via conventional echocardiography in both the DM and control groups (Table 1).”

should read:

“The morphology and function of the left ventricle (LV) were assessed via conventional echocardiography in both the DM and control groups (Table 1). Some data (including LA, IVS, LVPW, LVIDd, LVIDs, EF and FS) were republished from our previous article, Qiao et al.^1^.

Finally, an updated version of Table 1, with appropriate referencing is listed below.
